# A Novel Method for Extracting Glucomannan Using an Aqueous Two-Phase System

**DOI:** 10.17113/ftb.63.01.25.8583

**Published:** 2025-03

**Authors:** Enny Sholichah, Bambang Purwono, Agnes Murdiati, Akhmad Syoufian, Nok Afifah, Achmat Sarifudin

**Affiliations:** 1 Department of Chemistry, Faculty of Mathematics and Natural Science, Universitas Gadjah Mada, Jl. Sekip Utara, Bulaksumur, Yogyakarta, Indonesia; 2 Research Center for Appropriate Technology, National Research and Innovation Agency Jl. KS. Tubun No. 5 Subang, West Java, Indonesia; 3 Department of Food and Agriculture Product Technology, Faculty of Agriculture Technology Universitas Gadjah Mada, Jl. Flora No.1 Bulak sumur, Yogyakarta, Indonesia

**Keywords:** aqueous two-phase system (ATPS), glucomannan, ethanol/Na_2_HPO_4_ extraction, ethanol/K_2_HPO_4_ extraction, porang

## Abstract

**Research background:**

Glucomannan is a polysaccharide compound used widely in food and pharmaceutical industries. The tuber of *Amorphophallus muelleri* Blume is called porang in Indonesia. Ethanol extraction is commonly used to extract glucomannan from porang flour; however, the method has some limitations. Glucomannan obtained by ethanol extraction has a higher protein content than standard glucomannan. In this work, the salting-out effect of the salts of the aqueous two-phase system was investigated for the extraction of glucomannan. In this way, the protein can be removed from the glucomannan flour, thus increasing the purity of the obtained glucomannan.

**Experimental approach:**

A novel glucomannan extraction method using an aqueous two-phase system (ATPS) consisting of salt and ethanol was investigated. The Na_2_HPO_4_ and K_2_HPO_4_ salts at 3 different mass fractions (1, 2 and 3 %) mixed with 40 % ethanol in a 1:1 volume ratio were used to prepare the ATPS. The efficiency of ATPS in the extraction of glucomannan was based on the phase separation and better properties of glucomannan, including proximate composition, colour, thermal properties and surface morphology were obtained. Statistical analysis was performed to test the significant differences between the mean values of each treatment. The statistical significance level was set at p=0.05.

**Results and conclusions:**

The results showed that the aqueous two-phase system was able to separate a solution of porang flour into three layers, namely bottom (F1), middle (F2) and top (F3) layer. The bottom (F1) and middle (F2) layers were rich in glucomannan and starch, respectively, while the top layer (F3) consisted of an ethanol-soluble compound. The salts affected the yield of glucomannan and the properties of the obtained glucomannan, including colour, impurities (protein and ash), thermal properties, molecular mass and surface morphology. Increasing salt mass fraction decreased the yield of glucomannan but increased the yield of other components. ATPS reduced the protein content and increased the lightness of the glucomannan. The glucomannan obtained with ATPS had a higher thermal stability than the control.

**Novelty and scientific contribution:**

Salting-out ability of the salt of the aqueous two-phase system is commonly used in protein precipitation and isolation. However, there was no report found on the application of ATPS for the isolation of glucomannan. This study has shown that the ATPS (ethanol/Na_2_HPO_4_ and ethanol/K_2_HPO_4_) is a potential new extraction method for glucomannan extraction.

## INTRODUCTION

The tuber of *Amorphophallus muelleri* Blume, locally known as porang, is the most common source of glucomannan in Indonesia. Glucomannan is a polysaccharide molecule that is widely used as an ingredient in food and pharmaceutical industries. Glucomannan can be classified as a functional food because it forms short fatty acids in the intestinal system which improves immunity, assists in obesity therapies, regulates lipid metabolism, has a laxative effect, exhibits anti-diabetic, anti-inflammatory and prebiotic properties, and is also used as a wound dressing (*1*). The physicochemical properties of glucomannan are influenced by its degree of purity. Some global institutions such as the European Food Safety Authority, the Food and Agriculture Organisation (FAO) and the People's Republic of China, which are concerned with the quality of glucomannan, have issued standard requirements for glucomannan. The European Food Safety Authority recommends that glucomannan as a food additives should have protein and ash content less than 1.5 and 2 %, respectively (*2*). In addition, the FAO regulation states that konjac flour as a food additive should have a content of: glucomannan >75 %, protein <8 % and ash <5 % (*3*). The professional standard of the People's Republic of China for konjac flour also stipulates that the colour of glucomannan must be white in order not to impair its intended use (*4*). The main component of the porang tuber is glucomannan, but it also contains other components known as impurities, including starch, protein and ash (*5*, *6*). The glucomannan mass fraction on dry mass basis in some *Amorphophallus* tubers from Vietnam is about 5–9 % (*7*), while in the porang tuber it is about 16 %. The starch, protein and ash mass fraction of the porang tuber is 11.2, 4.28-9.5 and 0.83-5.69 %, respectively (*8*). The presence of proteins, starch and other polysaccharides reduce the viscosity of glucomannan. Ryan *et al.* (*9*) developed a method to reduce the viscosity of glucomannan for a beverage product by adding some compounds with different molecular mass such as dextrin, protein and hydrolysed guar gum. Therefore, glucomannan content is one of the other parameters that need to be analysed to classify the quality of porang flour (*10*). Research to obtain glucomannan that meets the glucomannan standards is still required.

Purification of glucomannan from other impure components has been carried out using mechanical and chemical methods. The mechanical separation method has been used to separate glucomannan from porang flour with a yield of 33.39-66.75 % and a glucomannan mass fraction of 47.45-60.67 % (*11*). In the chemical separation method, alcohol solvents, including ethanol and isopropyl alcohol, at various amounts and extraction temperatures have been successfully used to separate glucomannan from porang flour (*12*). Ethanol amount, extraction time and temperature are important factors when using ethanol for glucomannan extraction, as well as the number of extraction cycles. A glucomannan yield of 11.86-14.59 % was obtained with 50 % ethanol in 2 extraction cycles. The addition of extraction cycles increases the glucomannan content and decreases the amount of other components in the glucomannan product; however, it significantly increases the amount of wasted ethanol, extraction time and costs. On the other hand, the protein content of the glucomannan isolated using ethanol ranges from 3.8 to 5.18 % (*6*, *13*). Ultrasound-assisted extraction method has been used for polysaccharide extraction and it improved their biological activities (*14*). Our previous study indicated that a pretreatment involving freezing/thawing cycles could reduce the ash content of glucomannan but not its protein content and colour change (*15*). Therefore, a method to isolate glucomannan with low ash and protein content and acceptable colour needs to be developed.

Aqueous two-phase system (ATPS) shows an opportunity to separate biomacromolecules including glucomannan, starch, protein and organic colour compounds. The ATPS is a liquid-liquid extraction method that involves equilibrium, phase separation and solute concentration in one stage (*16*). The principle of ATPS extraction is the difference in solubility of a substance or material in a two-phase water system. ATPS can be made using a solution system consisting of polymer/polymer, polymer/salt, ionic compound/salt and salt/alcohol. The advantages of the ATPS are that it is more environmentally friendly, faster, easier to process and it produces glucomannan with a high yield, purity and production capacity (*17*). An ATPS consisting of ammonium sulfate and ethanol was used to extract polysaccharides from *Grifola frondosa*, *Selaginella doederleinii* and *Phellinus linteus* (*18*–*20*).

An ATPS formed by short-chain alcohols and salts shows many advantages, such as low viscosity, high mass transfer efficiency, stable and wide phase formation and it is cheaper than polymers (*21*). The optimum amount of ethanol for glucomannan extraction is 40-50 %. If it is less than 40 %, the granules of glucomannan can absorb more water molecules, dissolve and make a colloidal solution (*22*). Salt acts as an agent to form water-rich and ethanol-rich phases that can separate a component based on its solubility in water or alcohol (*23*). The selection of salt type affects the two-phase polarity and the salting-out effect. Furthermore, the salt and alcohol contents determine the formation of a two-phase solution (*24*). Phosphate salts are commonly used in the ATPS to fractionate polysaccharides and proteins (*25*). It has been reported that potassium hydrogen phosphate can induce the separation of water-ethanol molecules which are bound by hydrogen bonds (*23*) and it gives more salting-out to salting-in effect based on the anionic and cationic sequences (SO_4_^2-^<H_2_PO_4_^-^<Cl^-^<NO_3_^-^<ClO_4_^-^<SCN^-^ and K^+^<Na^+^<H^+^<Mg^2+^<Ca^2+^<Al^3+^) (*26*). However, no report on the implementation of ATPS for glucomannan isolation has been found. Therefore, this study investigates the isolation of glucomannan from porang flour using ATPS consisting of Na_2_HPO_4_/ethanol and K_2_HPO_4_/ethanol.

## MATERIALS AND METHODS

### Materials

The main material was porang flour, which was obtained from a local supplier in Subang, West Java, Indonesia. The chemical reagents were technical grade ethanol, analytical grade K_2_HPO_4_ (Merck, Damstadt, Germany), and Na_2_HPO_4_ (Merck, Damstadt, Germany), chromatography grade water (Merck, Damstadt, Germany) and standard polyethylene oxide/polyethylene glycol (Agilent, Craven Arms, UK).

### Process of glucomannan extraction using aqueous two-phase system

**Fig. S1:**
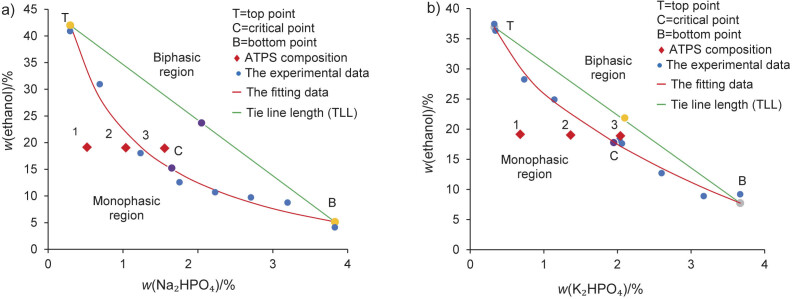
Binodal curve of the aqueous two-phase system (ATPS) composed of: a) ethanol/Na_2_HPO_4_ and b) ethanol/K_2_HPO_4_-ATPS. TLL=tie line length

The glucomannan was extracted based on the schematic diagram of the operation procedure of an extraction using alcohol/salt aqueous two-phase system (*27*). ATPS was made by mixing K_2_HPO_4_ and Na_2_HPO_4_ solution at the mass fractions of 1, 2 and 3 % with 40 % of ethanol at a volume ratio of 1:1. The salt mass fractions were selected based on the binodal curve of ethanol/K_2_HPO_4_ and ethanol/Na_2_HPO_4_ obtained in a preliminary study (Fig. S1 and Table S1). A mass of 100 g porang flour that passes through a 40-mesh screen was added to the 250 mL ATPS solution and then it was extracted in a high-speed blender (8010BU Set; Waring Blender Laboratory, Torrington, CT, USA) at 18 000 rpm for 2 min (Table S2). The preliminary study was conducted to compare the low-speed and high-speed mixing processes. The low-speed mixing process was conducted at 400 rpm for 30 min using a magnetic stirrer (SMHS-3; Daihan Scientific, Gangwon-do, South Korea). High-speed mixing was selected based on a preliminary study on the effect of low and high-speed mixing on the glucomannan yield (Table S2). After resting for 30 min, the solution was separated into 3 layers, with the extracted glucomannan in the bottom layer. The glucomannan extracted using ATPS will be referred to as the treated glucomannan in the subsequent discussion. The control glucomannan sample was prepared by extracting porang flour with 40 % ethanol.

### Separation of ATPS phases

Following the previous steps, porang flour in ethanol/K_2_HPO_4_ and ethanol/Na_2_HPO_4_ of ATPS was separated into three layers. The bottom layer (F1) and the middle layer (F2) were salt-rich phases and the top layer (F3) was ethanol-rich phase. The bottom layer and the middle layer were separated in a blender (Vitamax; Madato, Taipei, Taiwan) with a 60 mesh screen. The residue was mixed with 250 mL of 40 % ethanol, then mixed at a high speed mixer (8010BU set; Waring Blender Laboratory) and finally filtered again. This step was repeated with 70 % ethanol to wash the glucomannan fraction. After the washing step, the glucomannan fraction was dried at 50 °C for 12 h. Iodine test was used to confirm that starch component was separated from the glucomannan fraction. The filtrate obtained in the separation step was centrifuged (SL40R; Thermo Sciencetific, Osterode am Harz, Germany) at 2504×*g* for 30 min. The pellet was then dried to obtain starch fraction (F2). The yield of F1 and F2 was determined by dividing the mass of the dried F1 and F2 by the initial mass of porang flour. F3 was calculated by subtracting the percentage of F1 and F2 from 100 %. The control glucomannan sample was obtained according to the same protocol as the ATPS-treated samples.

### Determination of proximate composition of glucomannan

The proximate composition of the glucomannan includes moisture, ash, protein and carbohydrate content. The moisture and ash contents were assayed using a gravimetric method based on the determination of moisture and ash content in animal feed (*28*, *29*). Nitrogen combustion method was used to measure protein content using a protein analyzer (Büchi Dumaster D480; Elementar Analysensysteme, Langenselbold, Germany). The calculation of protein content used a nitrogen conversion factor of 5.7 according to the standards of the European Commission (*2*). Glucomannan content was calculated as the percentage of carbohydrates, which was determined by subtracting the sum of the percentages of total ash, moisture and protein content from 100 % (*3*).

### Colour

A spectrophotometer (CM700D; Konica Minolta, Osaka, Japan) was used to measure the colour of the glucomannan sample. The colour parameter of a sample was read in a cuvette. The data were reported including the value of L* (lightness index), a* (red to green index) and b* (yellow to blue index). The change in the colour of the treated sample was compared to the control and calculated using the following equation:


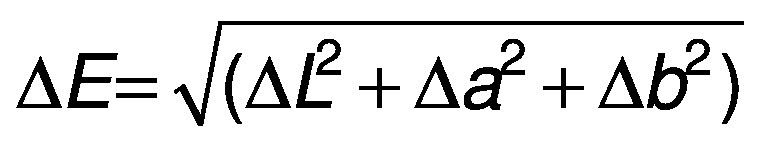
 /1/

### Fourier transform infrared spectroscopy

Fourier transform infrared (FTIR) spectrophotometer (Alpha II; Bruker, Ettlingen, Germany) was used to identify the functional groups of the obtained glucomannan. The analysis was carried out in the infrared range of 400-4000 cm^-1^ with a resolution of 4 cm^-1^.

### Nuclear magnetic resonance spectroscopy

The ^1^H NMR spectra were read on a nuclear magnetic resonance spectrometer (JNM ECZR500; Jeol, Tokyo, Japan). The sample was prepared according to Tang *et al.* (*30*) with modifications. A total of 40 mg of sample was dissolved in D_2_O (40 mg/mL) and mixed for 1 h, and the measurement was run at 25 °C and 500 MHz. Chemical shifts are expressed in ppm and tetramethylsilane was used as the reference standard. ^13^C CPMAS (solid NMR) of spectra were recorded on an NMR spectrometer (JNM-ECZ500R/S1 DPX200; Jeol) operating at a frequency of 125.76 MHz and used a solid-state probe equipped with 4 mm (o.d.) spinner. The spectra were recorded at 5000 scans, with relaxation delay 15 s and spin rate 10 kHz. The integration value of anomeric proton from ^1^H-NMR spectra was used to calculate the ratio of mannose and glucose. The results of the integration of anomeric carbon area at a chemical shift of 105 ppm and methyl carbon at 21 ppm were used to calculate the degree of acetylation (DA) using the following equation (*31*):


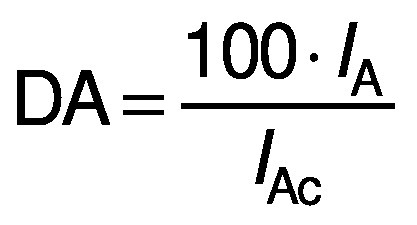
 /2/

where *I*_A_ is the integrated area of chemical shift at 105 ppm and *I*_Ac_ is the integrated area of chemical shift at 21 ppm.

### Molecular mass

The molecular mass of the glucomannan sample was determined using a gel permeation chromatography/size exclusion chromatography (GPC/SEC) system (1260 Infinity II; Agilent Technologies, Wadbronn, Germany) with a column PL 2080-0700 and two detectors, a refractive index (RI) detector and a viscometer detector. The mobile phase consisted of water and 0.02 % NaN_3_. The glucomannan sample was dissolved in water (1 mg/mL), stirred and filtered using 0.45-µL membrane filter (Agilent Captiva Econofilter; Agilent Technologies, Bejing, PR China). The flow rate of the eluent was 0.5 mL/min and the columns and detectors were maintained at 35 °C (*15*). Prior to use, the GPC/SEC was calibrated using standard substances of polyethylene oxide/polyethylene glycol (Agilent Technologies UK Ltd, Craven Arms, UK).

### Thermal analysis

The thermal properties of the sample were assessed by a differential thermal analysis-thermogravimetry (DTA-TG) apparatus (DTG-60; Shimadzu, Kyoto, Japan). The sample of 5 mg was placed on the aluminium sample pan, sealed and heated from 25 to 450 °C with an average heating rate of 10 °C/min (*7*).

### Morphological and residual mineral analysis using scanning electron microscopy with energy dispersive X-ray spectroscopy

The morphological properties of the glucomannan sample were observed by a scanning electron microscope (JSM-IT300LV; Jeol). Before analysis, the sample was sieved through an 80-mesh screen. The sample was placed on a metal stub that was previously covered with a double-sided adhesive tape. A rubber air blower pump dust cleaner was used to remove the excess sample from the metal stub. Prior to observation, the sample was coated with gold and it was examined with an accelerating voltage of 2 kV at magnification of 100, 500 and 1000 times.

### Statistical analysis

A completely randomized design (CRD) with 2 factors including the salt (Na_2_HPO_4_ and K_2_HPO_4_) and salt mass fraction of 1, 2 and 3 % was used as the experimental design. The effects of extraction using ATPS including the separation, proximate composition of glucomannan, colour and thermal properties were observed. Multivariate analysis of variance (MANOVA) followed by a *post-hoc* Duncan’s test were carried out to test the significant differences between the mean values of each treatment (*15*). The statistical significance level was set at p=0.05. The statistical analysis was conducted using SPSS v. 26 (*32*).

## RESULTS AND DISCUSSION

### ATPS method for glucomannan extraction

The aqueous two-phase system (ATPS) composed of ethanol, salt and water, where the binodal curves (Fig. S1 and Table S3) were constructed to determine the compositions of ethanol/Na_2_HPO_4_ and ethanol/K_2_HPO_4_, was used in this experiment. It shows that the binodal curve limited the two ATPS zones, namely monophasic region (lower side) and biphasic region (upper side). For the extraction 40 % ethanol (*22*) was chosen and then the mass fractions of 1, 2 and 3 % of salts (*18*) were selected based on their amount in the monophasic region and near the critical point of the binodal curve. Based on these results, the composition of ATPS used in this study is given in Table S1.

**Fig. S2:**
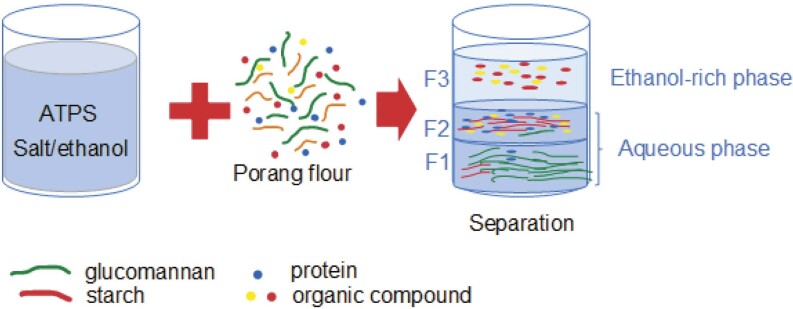
The proposed mechanism of glucomannan extraction using aqueous two-phase system (ATPS). F1=glucomannan, F2=starch and a water-soluble compounds, and F3=an ethanol-soluble compound

Fig. S2 shows the proposed mechanism of glucomannan extraction using the ATPS based on the visual observation during extraction. The extraction of glucomannan from porang flour using ATPS consisting of ethanol/Na_2_HPO_4_ and ethanol/K_2_HPO_4_ resulted in three layers, namely the bottom (F1), the middle (F2) and the top layer (F3). The bottom and middle layers (F1 and F2) were rich in salt-water phases. Based on the iodine testing (*5*), the colour of the bottom (F1) and top layer (F3) did not change, while the middle layer (F2) turned dark blue. This result indicated that the bottom layer was rich in glucomannan and the middle layer had a high starch content. The top layer (F3) was the ethanol-rich phase that contained many simple sugars, dyes and other compounds. The formation of three layers after the extraction was greatly influenced by the salts in the composition of ATPS. The K_2_HPO_4_ and Na_2_HPO_4_ are dissociated into K^+^, Na^+^ and HPO_4_^2-^ ions when they are dissolved in water. The ions can break the hydrogen bond between the water and the ethanol because of the hydration of ions (*33*). Hydration capacity of ions depends on the Gibbs energy of hydration ion (*25*). Furthermore, both salts have higher salting-out effect than other salts based on the ionic strength sequences of SO_4_^2−^<H_2_PO_4_^−^<Cl^−^<NO_3_^−^<ClO_4_^−^<SCN^−^ and K^+^<Na^+^<H^+^<Mg^2+^<Ca^2+^<Al^3+^ (*23*, *26*). When the salts are added to the glucomannan solution, the ions compete with the glucomannan molecules to bind the water. This phenomenon reduces the glucomannan solubility in the solution and eventually the glucomannan molecules precipitate in the bottom layer. A similar result was reported during polysaccharide extraction of *Lycium barbarum* L. with ATPS (*34*). The F1 and F2 (Fig. S2), which consisted of polysaccharides, were separated into two layers due to the difference in their water absorption capacity and molecular mass. The water absorption capacity of glucomannan, starch and cellulose is 50-100, 0.884-0.951 and 40 g/g, respectively (*5*, *35*, *36*).

The results of ATPS separation are shown in Table 1. Results indicated that the yield of glucomannan (F1) extracted using ATPS did not significantly change but was lower than that of the control. However, the results of the middle (F2) and the top layer (ethanol) (F3) were higher than that of the control. These might be because some glucomannan molecules were not separated from the starch in the middle layer (F2) since they have almost similar molecular mass of 10^6^ Da. Other possibility is that there was an interaction between glucomannan and the other polysaccharide molecules through an ion bridging mechanism. Glucomannan interacts with xanthan gum *via* Na^+^ and Ca^2+^ (*36*). In our experiment, ATPS with K_2_HPO_4_ yielded higher F2 than that with Na_2_HPO_4_. The K^+^ cation has a bigger atomic size than Na^+^, so they can have different ionic strength when they interact with starch and protein molecules according to the lyotropic sequence (*26*). Results also showed that increasing salt mass fraction up to 3 % increased the yield of F1 and F2. This might be due to the increase in the salting-out effect of the salt. F3 of the ATPS was higher than that of the control (ethanol extraction). It contained non-polar components such as flavonoids, polyphenols and ethanol-soluble alkaloids, as also reported by Wan *et al*. (*23*) and Xi *et al*. (*27*). Moreover, F3 also contained other organic compounds such as carotenoids, oligosaccharides and monosaccharides that were soluble in the ethanol phase.

**Table 1:** Result of separation in aqueous two-phase system (ATPS) extraction

**Sample**	***w*(F1_bottom part_)/%**	***w*(F2_middle part_)/%**	***w*(F3_top part_)/%**
**Control**	(54.4±3.07)^a^	(24.7±4.1)^a^	(20.9±7.1)^a^
**Ethanol/Na_2_HPO_4_ 1 %**	(51.2±2.9)^a^	(24.4±2.6)^a^	(24.4±5.5)^a^
**Ethanol/Na_2_HPO_4_ 2 %**	(44.3±5.2)^a^	(26.9±4.5)^a^	(28.8±0.7)^a^
**Ethanol/Na_2_HPO_4_ 3 %**	(5156±0.7)^a^	(23.7±4.4)^a^	(24.8±3.7)^a^
**Ethanol/K_2_HPO_4_ 1 %**	(46.6±9.1)^a^	(27.8±3.3)^a^	(25.6±5.8)^a^
**Ethanol/K_2_HPO_4_ 2 %**	(46.3±1.2)^a^	(27.8±0.2)^a^	(26.4±2.6)^a^
**Ethanol/K_2_HPO_4_ 3 %**	(49.0±0.6)^a^	(27.6±1.1)^a^	(22.9±2.4)^a^

The values marked with different letters in superscript in the same column are significantly different according to Duncan’s *post-hoc* test at a significance level of 5 %. F1=glucomannan, F2=starch and a water-soluble compound, and F3=ethanol-soluble compound. 1 to 3 %=mass fractions of salt in the solution

### Physicochemical properties

Results of proximate analysis (Table 2) indicated that the protein mass fraction of glucomannan extracted using ATPS was significantly lower than that of the control glucomannan (p<0.05). Cheng *et al*. (*37*) reported that protein could be denatured in the aqueous organic solvents and precipitated in the aqueous phase. Furthermore, the salting-out effect of K_2_HPO_4_ and Na_2_HPO_4_ caused the decrease of solubility of the protein in water and it was precipitated in the middle layer (Table 3). Therefore, the protein mass fraction of glucomannan obtained from the ATPS was lower than that obtained by the conventional extraction method (control). These results were similar to the report of Antunes *et al*. (*25*) where the separation of polysaccharides using ATPS resulted in the lower protein content. The ATPS extraction effectively reduced the protein content up to <0.5 %. Thus, the protein mass fraction obtained from this study was lower than that obtained by ethanol extraction (3.8-4.4 %) (*13*), freeze-thaw cycle pre-treatment (1.4-2.3 %) (*15*) and microwave-assisted extraction (0.82 %) (*38*).

**Table 2:** Proximate content on dry mass basis of glucomannan obtained from ethanol extraction (control) and aqueous two-phase system (ATPS) extraction

**Sample**	***w*(moisture)/%**	***w*(ash)/%**	***w*(protein)/%**	***w*(glucomannan)/%**
**Porang flour**	(7.4±0.6)	(4.1±0.6)	(9.7±2.8)	(78.4±1.0)
**Control**	(7.2±0.9)^b^	(0.79±0.01)^b^	(1.0±0.2)^b^	(91.0±0.)^cd^
**Ethanol/Na_2_HPO_4_ 1 %**	(13.0±0.4)^d^	(0.4±0.2)^a^	(0.5±0.1)^a^	(86.01±0.6)^b^
**Ethanol/Na_2_HPO_4_ 2 %**	(8.52±0.04)^c^	(0.41±0.07)^a^	(0.43±0.01)^a^	(90.6±0.1)^c^
**Ethanol/Na_2_HPO_4_ 3 %**	(13.9±0.1)^d^	(1.3±0.2)^bc^	(0.51±0.01)^a^	(84.3±0.3)^a^
**Ethanol/K_2_HPO_4_ 1 %**	(8.52±0.00)^a^	(0.75±0.02)^ab^	(0.6±0.1)^a^	(90.2±0.1)^c^
**Ethanol/K_2_HPO_4_ 2 %**	(6.0±0.2)^a^	(1.5±0.6)^cd^	(0.57±0.00)^a^	(91.4±0.4)^d^
**Ethanol/K_2_HPO_4_ 3 %**	(7.0±0.1)^b^	(2.0±0.3)^d^	(0.58±0.07)^a^	(90.3±0.3)^c^

The values with different letters in superscript in the same column are significantly different according to Duncan’s *post-hoc* test at a significance level of 5 %. 1 to 3 %=mass fractions of salt in the solution

**Table 3:** Sodium, potassium and phosphorus mass fraction of F1_bottom layer_ observed by scanning electron microscopy combined with energy dispersive X-ray analysis (SEM-EDX) and protein of F2_middle layer_

	F1_bottom layer_			F2_middle layer_
Sample	*w*/%			*w*(protein)/%
	Na	K	P	
Ethanol/Na_2_HPO_4_ 1 %	0.35	0.13	0.19	(7.89±0.05)^c^
Ethanol/Na_2_HPO_4_ 2 %	0.42	0.21	0.21	(7.92±0.01)^c^
Ethanol/Na_2_HPO_4_ 3 %	0.26	0.11	0.10	(6.4±0.1)^a^
Ethanol/K_2_HPO_4_ 1 %	0	0.66	0.18	(9.7±0.1)^e^
Ethanol/K_2_HPO_4_ 2 %	0	0.97	0.27	(8.60±0.01)^d^
Ethanol/K_2_HPO_4_ 3 %	0	1.07	0.31	(7.43±0.04)^b^

F1=glucomannan fraction at the bottom layer and F2=starch and a water-soluble compound at the middle layer. 1 to 3 %=mass fractions of salt in the solution

Results (Table 2) also showed that the ash mass fraction of glucomannan extracted by ethanol/Na_2_HPO_4_ was lower than that obtained using ethanol/K_2_HPO_4_. This result indicated that glucomannan contained a residue of potassium and phosphate from the ATPS. The results obtained by scanning electron microscopy combined with energy dispersive X-ray analysis (SEM-EDX) showed that the mass fractions of potassium and phosphorus in the glucomannan obtained by ethanol/K_2_HPO_4_ extraction (Table 3) were higher than those obtained by ethanol extraction. The K^+^ may have higher ionic interaction with glucomannan molecules than the Na^+^ because the atomic size of K^+^ is larger than of Na^+^. The degree of ionic binding is directly related to the nuclear charge effect that depends on the size and charge of the dissolved ions (*39*).

Table 4 shows that glucomannan extracted using ATPS was brighter in colour than that of the control (p<0.05). Results indicated that the lightness values of glucomannan obtained by ATPS increased significantly compared to those of the control. Moreover, the total colour difference (Δ*E*) showed that the treated samples were different from the control. Results also showed that higher salt percentages of ATPS caused lower lightness values of glucomannan. Among the treated samples, glucomannan produced using 2 % Na_2_HPO_4_ had the highest lightness value and the lowest values of a* and b* parameters and the highest Δ*E* value. ATPS can inhibit browning reactions by inhibiting mechanisms of the activity of oxidizing enzymes (*22*). Moreover, the addition of salt affected the increasing polarity of the bottom layer, which resulted in an increase in the solubility of organic compounds in the ethanol-rich layer (top layer) including the carotenoid compounds. The colour of glucomannan is influenced by the natural yellow-orange colour characteristics of porang tuber (*5*). Porang tuber contains organic compounds such as carotenoids, polyphenols and other colour compounds that are susceptible to oxidation reactions. The oxidizing reaction occurs more intensively during the processing stages of porang chip and flour, particularly when the sliced porang tubers are exposed to air (*40*). Therefore, the colour of glucomannan obtained by ATPS had higher lightness and lower a* and b* values.

**Table 4:** Colour and molecular mass of glucomannan obtained by ethanol extraction (control) and aqueous two-phase system (ATPS) extraction

**Sample**	L*	a*	b*	***ΔE***	***M*_w_*/*(g/mol)**	***M*_n_*/*(g/mol)**	**PDI**
**Control**	(73.57±0.00)^a^	(4.53±0.02)^g^	(10.48±0.00)^c^	0	(2.25·10^6^±1.34·10^5^)^b^	(7.48·10^5^±2.97·10^4^)^ab^	(3.0±0.3)^ab^
**Ethanol/Na_2_HPO_4_ 1 %**	(79.16±0.00)^f^	(3.25±0.01)^c^	(10.30±0.01)^b^	(15.88±0.05)^d^	(1.55·10^6^±5.94·10^5^)^ab^	(6.70·10^5^±8.49·10^4^)^ab^	(2.3±0.6)^a^
**Ethanol/Na_2_HPO_4_ 2 %**	(80.80±0.01)^g^	(2.71±0.00)^a^	(9.56±0.01)^a^	(26.57±0.07)^e^	(2.30·10^6^±2.12·10)^5b^	(9.95·10^5^±4.95·10^4^)^b^	(2.4±0.3)^ab^
**Ethanol/Na_2_HPO_4_ 3 %**	(78.14±0.01)^d^	(3.85±0.01)^d^	(10.89±0.01)^d^	(11.43±0.03)^c^	(2.07·10^6^±4.03·10^5^)^ab^	(7.28·10^5^±1.68·10^5^)^ab^	(2.9±0.1)^ab^
**Ethanol/K_2_HPO_4_ 1 %**	(79.11±0.01)^e^	(3.11±0.00)^b^	(10.29±0.00)^b^	(15.92±0.01)^d^	(2.9·10^6^±3.11·10^5^)^a^	(6.38·10^5^±3.29·10^5^)^ab^	(2.5±0.4)^ab^
**Ethanol/K_2_HPO_4_ 2 %**	(75.49±0.00)^b^	(4.18±0.00)^f^	(11.89±0.01)^f^	(2.77±0.01)^a^	(1.8·10^6^±3.61·10^5^)^ab^	(7.47·10^5^±2.07·10^5^)^ab^	(2.8±0.4)^ab^
**Ethanol/K_2_HPO_4_ 3 %**	(76.18±0.01)^c^	(4.01±0.01)^e^	(11.46±0.01)^e^	(3.57±0.04)^b^	(1.7·10^6^±9.19·10^4^)^ab^	(5.42·10^5^±6.72·10^4^)^a^	(3.4±0.6)^b^

The values marked with different letters in superscript in the same column are significantly different according to Duncan’s *post-hoc* test at a significance level of 5 %. 1 to 3 %=mass fractions of salt in the solution, *M*_w_=average molecular mass, *M*_n_=number of average molecular mass, PDI=polydispersity index

### Structural properties

Results showed that extraction using ATPS produced glucomannan with the average molecular mass (*M*_w_) ranging from 1.55·10^6^ to 2.9·10^6^ g/mol and the number of average molecular mass (*M*_n_) was 5.42·10^5^-9.95·10^5^ (Table 4). This result indicated that the molecular mass of glucomannan was not affected by the ATPS extraction method (p>0.05). Jiang *et al.* (*41*) reported that salt did not affect the degradation of molecular mass of the polysaccharides. The molar mass (*M*) of glucomannan depends on the species of *Amorphophallus*. For instance, the molar mass of glucomannan isolated from *Amorphophallus paeoniifolius, Amorphophallus panomensis*, *Amorphophallus tonkinensis* and *Amorphophallus konjac* is 1.115·10^6^, 1.023·10^6^, 1.043·10^6^ and 9.1·10^5^ g/mol, respectively (*7*, *13*).

Table 4 shows that the isolated glucomannan has a polydispersity index (PDI) ranging from 2.27 to 3.35, which was similar to previous studies (*5*, *42*). This result showed that isolated glucomannan has a broad molecular mass distribution. It also indicated that the synthesis of glucomannan occurred by an uncontrolled reaction mechanism, namely chain reaction. Due to chain reaction mechanism, polymer chains are formed with widely varying molecular mass indicated by PDI between 1.5 and 20 (*43*). Qi *et al*. (*44*) reported that the biosynthesis pathway of glucomannan in plant occurred by enzymatic mechanisms that produce glucomannan molecules with varying chain lengths.

**Fig. 1:**
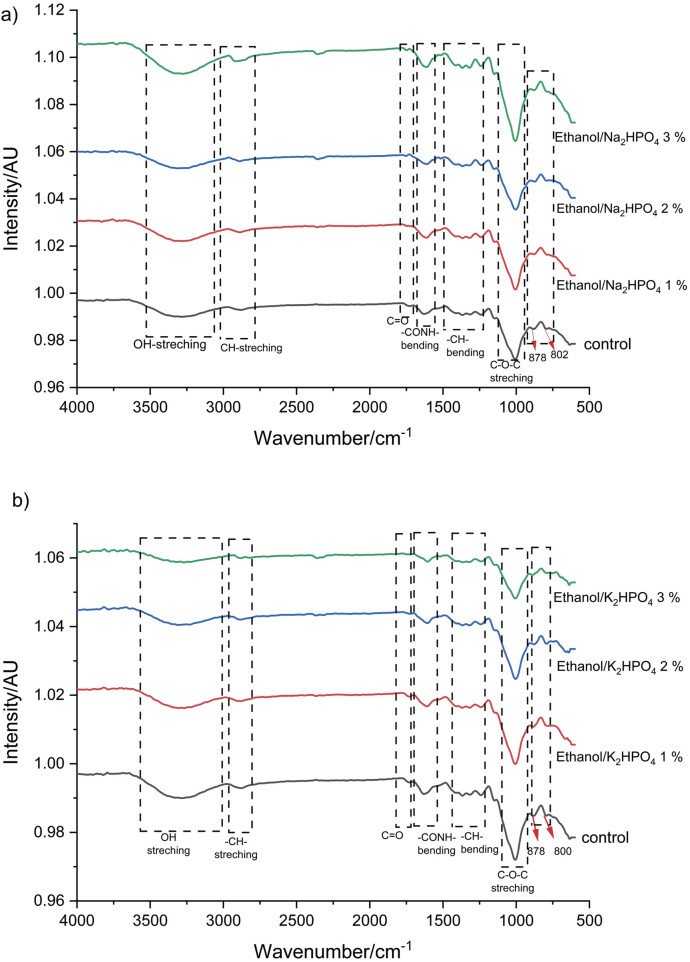
Fourier transform infrared spectra of glucomannan: a) glucomannan obtained by ethanol/Na_2_HPO_4_ extraction, and b) glucomannan obtained by ethanol/K_2_HPO_4_ extraction. 1 to 3 %=mass fractions of salt in the solution

The FTIR spectra of glucomannan obtained by ATPS (Fig. 1) show the groups of glucomannan structure. The wide peak at 2900-3600 cm^-1^ is a typical peak of the OH group originating from glucomannan monomers, both glucose and mannose. In addition, the broad peak indicates a large number of hydrogen bonds or bound water molecules. The -CH- aliphatic, C=O of the acetyl, C-H bending and C-O-C group appear at 2800-2900, 1724, 1200-1400 and 1000-1100 cm^-1^, respectively. The protein content, as another component of the glucomannan, was detected by the presence of the amide group peak -CONH- at a wavenumber of 1640 cm^-1^. The FTIR spectra of the obtained glucomannan showed the same pattern as those of the glucomannan obtained by extraction with ethanol (*45*). The bound water in the glucomannan was identified by the presence of peaks at 1611 and 1411 cm^-1^ (*30*). The peaks at 878 and 800 cm^-1^ were attributed to β-glucosidic and β-mannosidic linkages, respectively. This result was in agreement with the glucomannan extracted from *Amorphophallus konjac* (*13*).

**Fig. 2:**
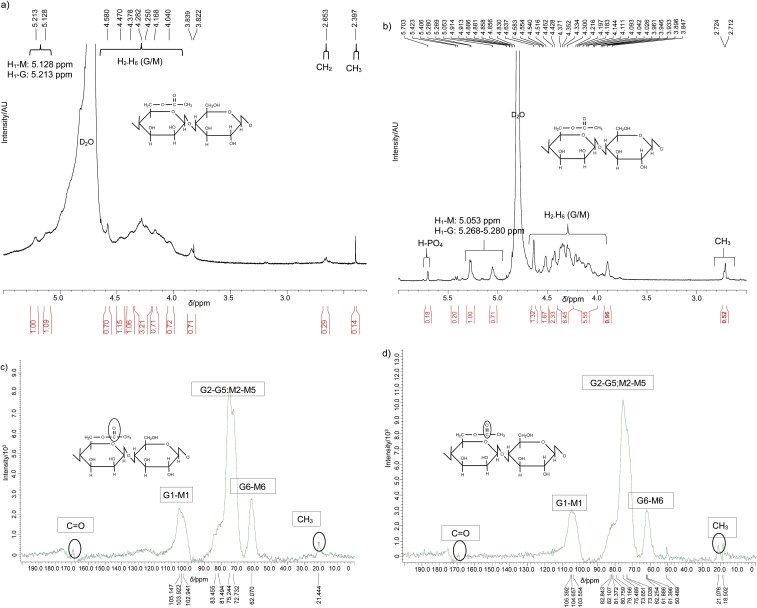
NMR spectra of glucomannan: a) ^1^H NMR of control, b) ^1^H NMR of glucomannan obtained by aqueous two-phase system (ATPS) extraction, c) ^13^C-NMR of control glucomannan, and d) ^13^C-NMR of glucomannan obtained by ATPS extraction

NMR spectra of glucomannan showed the chemical shift (*δ*) of the proton and carbon of the glucomannan. The proton and carbon spectrum patterns of glucomannan were identical to those of control glucomannan (Fig. 2). The difference was in the appearance of a proton from the salt residue (Na_2_HPO_4_ or K_2_HPO_4_) at a *δ*=5.703 ppm. The chemical shift of anomeric proton (H1) of glucomannan control was observed at *δ*=5.128 ppm for H1 mannose and 5.213 ppm for H1 glucose (Fig. 2a). The protons (H2-H6) were at *δ*=3.822-4.580 ppm. Anomeric proton of the treated glucomannan (Fig. 2b) showed the chemical shift at *δ*=5.053 and 5.268-5.280 ppm for H1-mannose and glucose, respectively. Meanwhile, the proton shift from H2 to H6 (mannose/glucose) was 3.933-4.637 ppm and the proton of methyl group

(-CH_3_) of acetyl appeared at *δ*=2.7 ppm. Enomoto-Rogers *et al.* (*46*) reported that 1.9, 2.0 and 2.1 ppm are the chemical shift values for the proton of the acetyl group and the chemical shift was 3.3-4.1 ppm for H2-H6 protons from polysaccharides (*47*). The ^1^H NMR spectra of control and treated glucomannan were similar to a previous study (*48*).

The ratio of glucose and mannose in the glucomannan was calculated by the ratio of the integration of H1 of glucose and mannose. The control and treated glucomannan had mannose/glucose ratios of 1.09:1.00 and 0.71:1.0, respectively. Meanwhile, the ratio of H1 mannose/glucose of glucomannan extracted from *Amorphopallus panomensis* in Vietnam and *Amorphophallus konjac* was 1.00:0.13 and 1.6:1.0, respectively (*49*, *50*). These results show that the *Amorphophallus* species influenced the chemical structure of glucomannan (*42*).

The ^13^C NMR spectra of the anomeric carbon of control and treated glucomannan were 102.941-105.147 ppm (Fig. 2c) and 103.554-105.392 ppm (Fig. 2d). The chemical shift of C2-C5 is 50.489-82.843 ppm with overlapping peaks indicating that the carbon atoms of the pyranose ring, namely glucose and mannose, have almost the same properties (*50*). C6 has a chemical shift at 62.070 ppm for control glucomannan and 61.396-62.254 ppm for the treated glucomannan. The acetyl group appeared as C=O at chemical shift at 170 ppm and CH_3_ at 21 ppm. The α- and β-glucose and mannose configuration can be determined using the chemical shift of anomeric proton (H1)/carbon (C1) at 90–110 and 4.5–5.5 ppm (*30*). Chemical shift of anomeric carbon at 98-108 and 101-105 ppm indicated the α-glycoside or β-glycoside bonds, respectively (*48*). According to the chemical shift of anomeric carbon, the β-glycoside bonds contructed both glucomannan structures. The involvement of C4 in the formation of glycosidic bonds is shown at a chemical shift of 79.24 ppm (*49*, *51*). Therefore, based on the proton and carbon shift values, the α-glycoside bonds formed are β(1→4)-glycoside and β(1→6)-glycoside. These bonds indicate that the structure of glucomannan has a straight chain as a backbone and a branched structure (*48*).

The peaks for acetyl carbon -CH_3_ and C=O at chemical shifts of 21 and 170 ppm, respectively, were of low intensity. The ratio of peak areas at 21 and 105 ppm indicated the degree of acetylation (DA) of glucomannan (*31*). The results showed that the DA of control glucomannan and the treated glucomannan was 4.46 and 1.88. The reducing value of DA of the treated glucomannan due to deacetylation process was due to the interaction with Na_2_HPO_4_ salt (*52*).

### Thermal properties

**Fig. 3:**
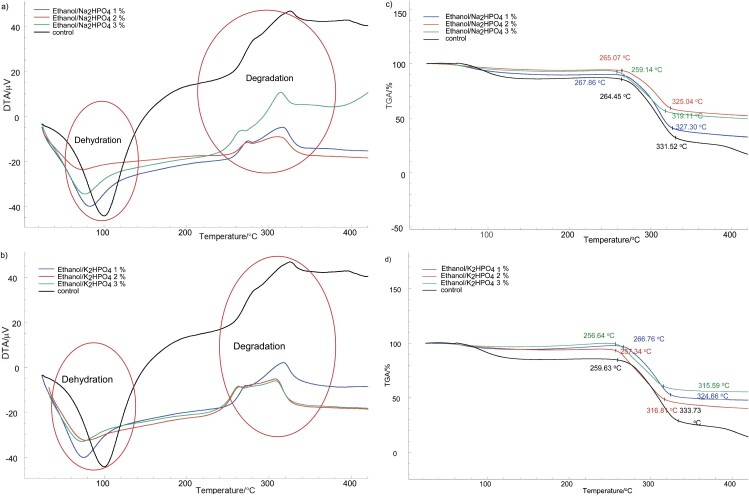
Determination of thermal properties: a) differential scanning calorimetry (DSC) thermogram of control glucomannan and the glucomannan obtained by ethanol/Na_2_HPO_4_ extraction, b) DSC thermogram of control glucomannan and glucomannan obtained by ethanol/K_2_HPO_4_ extraction, c) thermogravimetric analysis (TGA) of control glucomannan and glucomannan obtained by ethanol/Na_2_HPO_4_ extraction, and d) TGA of control glucomannan and glucomannan obtained by ethanol extraction/K_2_HPO_4_. 1 to 3 %=mass fractions of salt in the solution

Results indicated that glucomannan extraction using ATPS influenced the thermal properties of the obtained glucomannan (Fig. 3). The thermogravimetric analysis (TGA) and differential scanning calorimetry (DSC) thermograms of all samples showed consistent thermal degradation patterns of glucomannan in which the first and second peaks show dehydration and degradation patterns, respectively. The dehydration process of the control glucomannan required higher energy and occurred at higher temperature than that of the treated samples (Table S4). Fig. 3a, Fig. 3b and Table S4 show that the onset temperature of degradation of the control glucomannan was higher than that of the glucomannan from the ATPS. This indicated that the glucomannan extracted using ATPS was easier to degrade than the control glucomannan at the starting point. The protein content of the control glucomannan was higher than that of the treated samples. Protein and glucomannan can interact through hydrogen bond between the hydroxyl group (-OH) and the amine group (-NH-) (*53*), an interaction that can enhance the thermal stability of protein.

Thermogravimetric analysis (Fig. 3c, Fig. 3d and Table S5) showed that the mass loss of the control glucomannan was higher than of the treated samples. This result indicated that the phosphate residue may act as the stabiliser of glucomannan molecules, which is similar to the previous results in that there was an effect of salt in potato starch and iota-carrageenan solutions (*54*). Deng *et al*. (*55*) reported that the leftover phosphate in the glucomannan sample prevented mass loss during the degradation step.

### Morphological properties

**Fig. 4:**
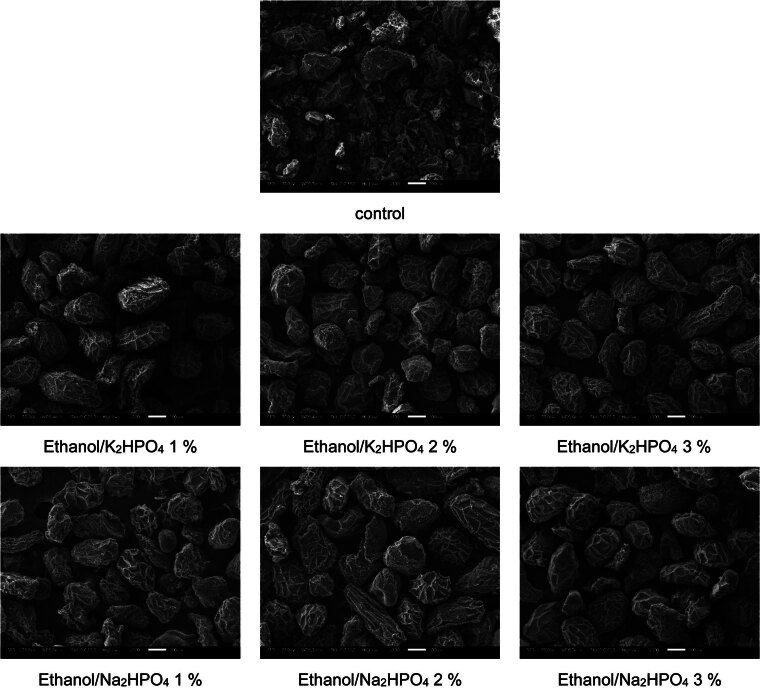
Morphological surface of the control glucomannan and glucomannan extracted using aqueous two-phase system ATPS) observed with 100× magnification. 1 to 3 %=mass fractions of salt in the solution

Fig. 4 shows the morphological surface of glucomannan particles. The morphological surface of the particles of the control glucomannan was different from that of the treated samples. The particles of the treated samples were relatively uniform in size compared to the particles of the control glucomannan. The morphological surface of control glucomannan was similar to that of the purified konjac glucomannan as reported by Yanuriati *et al*. (*5*). However, there were no significant differences among the particles of the treated samples. This result emphasised that the extraction using ATPS was able to produce uniform glucomannan particles and the extraction method did not destroy them. The surface of the glucomannan particles was wrinkled. Some impurities can be trapped on the surface, including starch, cellulose, protein and soluble sugar (*56*). The presence of the phosphate group might result in glucomannan particles with rougher surfaces and larger size (*55*).

## CONCLUSIONS

Aqueous two phase system (ATPS) is a new green method for the isolation of glucomannan from porang (*Amorphophallus muelleri* Blume) flour compared to the conventional glucomannan isolation using ethanol extraction. Using ATPS, a solution of porang flour was separated into three layers, namely the bottom layer containing glucomannan, the middle layer containing starch and other water-soluble compounds, and the top layer containing ethanol-soluble compounds. The ATPS method produced glucomannan with a brighter colour, lower protein content and more stable thermal properties than the control sample. The ATPS with higher salt content was able to produce glucomannan with lower lightness values. The glucomannan particles with uniform shapes were observed by SEM-EDX. The glucomannan obtained by ATPS using Na_2_HPO_4_ had better properties in terms of ash and protein content, colour, molar mass, polydispersity index and thermal properties than those obtained with K_2_HPO_4_. Therefore, ATPS using Na_2_HPO_4_, ethanol and water mixture proved to be a promising new method for the extraction of glucomannan. We recommend optimising the scalling up of ATPS extraction for future research before its industrial application for glucomannan extraction.

## Appendix

**Table S1:** Composition of the aqueous two-phase system (ATPS)

**ATPS**	***w*(salt)/%**	***w*(water)/%**	***w*(ethanol)/%**
Ethanol/Na_2_HPO_4_ 1 %	0.52	80.32	19.16
Ethanol/Na_2_HPO_4_ 2 %	1.04	79.90	19.07
Ethanol/Na_2_HPO_4_ 3 %	1.56	79.48	18.97
Ethanol/K_2_HPO_4_ 1 %	0.68	80.19	19.13
Ethanol/K_2_HPO_4_ 2 %	1.36	79.63	19.00
Ethanol/K_2_HPO_4_ 3 %	2.04	79.08	18.88

1 to 3 %=mass fractions of salt in the solution

**Table S2:** The effect of low and high speed mixing on the extraction of glucomannan from porang flour

	Yield/%				Glucomannan-proximate composition					Starch			
					*w*/%								
Porang flour	Glucomannan		Impurity		Glucomannan		Impurity		Glucomannan			Impurity	
	Low speed	High speed	Low speed	High speed	Low speed	High speed	Low speed	High speed	Low speed		High speed	Low speed	High speed
Mesh 60	(63.3± 1.1)^aA^	(59.4± 1.0)^bB^	(13.2± 0.4)^a^	(22.4±1.7)^a^	(71.0±24)^aB^	(80.66± 3.58)^aA^	(1.7± 0.3)^aB^	(4.1±0.2)^a^	(20.7±0.6)^b^		(6.5±0.5)^b^	(28.4±0.7)^b^	(31.4±0.5)^b^
Mesh 80	(63.6± 1.3)^a^	(62.0±1.0)^a^	(12.9± 0.5)^a^	(21.9±1.6)^a^	(70.4±6.4)^aA^	(80.20± 0.08)^aA^	(1.3± 0.7)^aA^	(0.9±0.3)^cC^	(22.2±1.0)^aA^		(7.1±0.5)^abAB^	(28.5±0.3)^bB^	(32.1±0.5)^abAB^
Mesh 100	(65.8± 1.3)^a^	(62.7±0.7)^a^	(12.80± 0.09)^a^	(20.6±1.5)^a^	(67±0.04)^bB^	(78.060.81)^aA^	(0.7±1.5)^aB^	(1.8±0.2)^bB^	(22.7±0.8)^a^		(8.0±0.5)^a^	(31.0±0.3)^a^	(33.2±1.0)^a^

Low speed: 400 rpm for 30 min, high speed: 18 000 rpm for 2 min

**Table S4:** DSC peaks of glucomannan obtained from ethanol extraction (control) and the aqueous two-phase system (ATPS) extraction

	Peak 1		Peak 2		Peak 3	
Sample	Temperature/°C	Δ*H*/mcal	Temperature /°C	Δ*H*/mcal	Temperature/°C	Δ*H*/mcal
Control	99.87	- 3060	293.09	56.94	325.58	223.18
Ethanol/Na_2_HPO_4_ 1 %	83.19	-1310	274.87	27.83	316.37	280.99
Ethanol/Na_2_HPO_4_ 2 %	73.74	-402.89	273.63	0.33	310.95	190.10
Ethanol/Na_2_HPO_4_ 3 %	76.72	-1080	268.23	9.41	314.32	278.49
Ethanol/K_2_HPO_4_ 1 %	75.28	-1690	274.21	34.39	317.40	313.19
Ethanol/K_2_HPO_4_ 2 %	77.97	-613.11	264.56	34.74	307.71	124.75
Ethanol/K_2_HPO_4_ 3 %	72.69	-770.57	262.73	47.22	307.61	162.77

1 to 3 %=mass fractions of salt in the solution

**Table S5:** TGA thermogram of glucomannan obtained from ethanol extraction (control) and the aqueous two-phase system (ATPS) extraction

**Sample**	**Temperature/°C**	***w*(mass loss)/%**
**Control**	299.63-.73	55.69
**Ethanol/Na_2_HPO_4_ 1 %**	267.86-327.30	47.92
**Ethanol/Na_2_HPO_4_ 2 %**	265.07-325.04	34.28
**Ethanol/Na_2_HPO_4_ 3 %**	259.14-319.11	36.14
**Ethanol/K_2_HPO_4_ 1 %**	266.76-324.66	44.18
**Ethanol/K_2_HPO_4_ 2 %**	257.34-316.81	44.49
**Ethanol/K_2_HPO_4_ 3 %**	256.64-315.59	38.64

1 to 3 %=mass fractions of salt in the solution

**Table S3:** The tie line length (TLL) and critical point of aqueous two-phase system (ATPS)

		C-point	
Salt	TLL	X (*w*(salt)/%)	Y (*w*(ethanol)/%)
Na_2_HPO_4_	37.02	1.65	15.27
K_2_HPO_4_	29.39	1.95	17.79
